# Exploring the Burden of PM2.5-Related Deaths and Economic Health Losses in Beijing

**DOI:** 10.3390/toxics12060377

**Published:** 2024-05-21

**Authors:** Xiaoqi Wang, Bart Julien Dewancker, Dongwei Tian, Shao Zhuang

**Affiliations:** 1 Faculty of Environmental Engineering, The University of Kitakyushu, Kitakyushu 808-0135, Japan; qi1981@163.com; 2 School of Architecture and Urban Planning, Beijing University of Civil Engineering and Architecture, Beijing 100044, China; tiandongwei77@163.com; 3 School of Landscape Architecture, Beijing Forestry University, Beijing 100083, China; no81terry@bjfu.edu.cn

**Keywords:** fine particulate matter, public health, mortality burden, economic value

## Abstract

Air pollution is one of the major global public health challenges. Using annual fine particulate matter (PM2.5) concentration data from 2016 to 2021, along with the global exposure mortality model (GEMM), we estimated the multi-year PM2.5-pollution-related deaths divided by different age groups and diseases. Then, using the VSL (value of statistical life) method, we assessed corresponding economic losses and values. The number of deaths attributed to PM2.5 in Beijing in 2021 fell by 33.74 percent from 2016, while health economic losses would increase by USD 4.4 billion as per capita disposable income increases year by year. In 2021, the average annual concentration of PM2.5 in half of Beijing’s municipal administrative districts is less than China’s secondary ambient air quality standard (35 μg/m^3^), but it can still cause 48,969 deaths and corresponding health and economic losses of USD 16.31 billion, equivalent to 7.9 percent of Beijing’s GDP. Therefore, it is suggested that more stringent local air quality standards should be designated to protect public health in Beijing.

## 1. Introduction

Air pollution is one of the major challenges facing global public health [1]. In 2022, according to the Global Burden of Disease (GBD), a study revealed that air pollution ranked fourth among various risk factors [2]. Fine particulate matter (PM_2.5_) pollution is the main pollutant in urban atmosphere. PM_2.5_ refers to dust or drifting dust with a diameter of 2.5 μm or less in ambient air. Atmospheric PM_2.5_ pollution poses a grave public health risk, and the death toll is increasing. The Global Burden of Disease study indicated that PM_2.5_ exposure led to approximately 6.67 million additional deaths worldwide and 1.42 million deaths in China in 2019 [3]. Therefore, addressing air pollution as a public health issue is one of the most urgent tasks facing the world [4], and quantifying and accurately assessing the death burden and economic loss related to PM_2.5_ is one of the important tasks [5].

The impact of pollutant concentrations on public health has long been part of the focus of the WHO (World Health Organization). A precise and comprehensive evaluation of the health effects associated with pollutant concentrations remains a top priority for the WHO. Additionally, the accurate assessment of mortality rates and economic losses attributable to PM_2.5_ pollution is of utmost importance. In the field of epidemiology, many models, such as the IER (integrated exposure–response) model [6], the NLP (non-linear programming) model [7], and the LL model (log–linear programming) [8], are used to estimate cause-specific mortality. However, these models have many uncertainties; for example, they do not evaluate the health hazards related to both indoor and outdoor PM2.5 pollution differently. Compared to other models, the GEMM model initiated by Burnett et al. (2018) [9] takes into account a wider range of health endpoints, considers indoor and outdoor sources of pollution, ADAPTS data, and specific conditions in different regions, and maintains high accuracy and relevance in applications in different regions of the world [10,11]. Therefore, this study utilized the GEMM model to assess the long-term health impacts of PM_2.5_ pollution in 16 districts in Beijing. Recent research findings have shown the health impact assessment of PM_2.5_ by using ground monitoring data combined with pollution exposure mortality models at national and urban agglomeration scales. Bell et al. (2007) conducted research about the influence of temporal and spatial differences within the chemical composition of PM_2.5_ on health in the United States, and the results showed that the daily changes in the composition of PM_2.5_ had an important impact on health [12]. Song et al. (2016) discussed the distribution features of PM_2.5_ in China’s air environment in 2013 and explored the health effects of PM_2.5_ by taking four health endpoints of PM2.5 [13]. Yin et al. extracted the annual PM_2.5_ concentrations of 16 districts in Beijing in 2012, estimated the external cost by using the statistical life value method (VSL) and the revised human capital method (AHC), and found that among all health effects, the economic losses caused by PM_2.5_-related deaths took up more than four-fifths of the total external cost [14].

In response to the negative effects caused by air pollution in cities, the Chinese government and local governments have implemented and updated standards of air quality and conducted specific control measures since 2013, when the state council initiated the Air Pollution Prevention and Control Action Plan [15]. While some studies report improvements in air quality and public health outcomes, others highlight persistent challenges, such as enforcement issues, regional disparities, and the need for more stringent standards. Today, more and more evidence proves that low concentrations of PM_2.5_ still cause health damage, and the goal of reducing PM_2.5_ concentration is urgent. Therefore, there is an urgent need for an effective evaluation method of PM_2.5_ pollution, which can offer essential information for regional policy goals, assess the public health impact of coordinating air pollution measures and reducing PM_2.5_ emissions, and identify potential economic benefits, to further develop scientific and economic reduction strategy.

Previous regional studies in China [13,14,15,16,17,18] mostly used low-resolution PM_2.5_ concentration values as exposure and mostly estimated PM_2.5_-attributed death relying on the comprehensive exposure–response model, non-linear model, log–linear model, and other models, but there are many uncertainties in the above models. Compared with the IER model, the GEMM proposed by Burnett et al. (2018) may take a more refined approach and data in assessing exposure, allowing them to more accurately capture changes and impacts in exposure levels. The GEMM combines data and results from multiple studies to create a more comprehensive assessment framework with some flexibility to adapt and modify for different regions and research needs. This flexibility allows GEMM to adapt to different environments and study conditions to provide more accurate and applicable health risk assessment results. The current commonly used health assessment model is the classical global burden of disease integrated risk function (GBD-IER) [16], while the recently proposed global exposure mortality model (GEMM) is less commonly used. This study seeks to address these limitations by proposing an alternative approach that combines high-resolution PM_2.5_ concentration data with a refined analysis of health outcomes, thereby offering a more accurate and comprehensive assessment of the health impacts of PM_2.5_ pollution.

The terrain in Beijing is relatively flat, but the contradiction between air pollution control and economic development is prominent. As the capital of China, the complex urban heat island effect in Beijing makes the transport of air and pollutants more complicated. It is one of the cities with serious air pollution in China, and haze has become one of the highly concerned issues among Beijing residents. Although the annual average concentration of PM_2.5_ in Beijing improved in recent years, there is still a significant gap compared to developed countries and regions [17]. At the same time, with the worsening of the aging population, the burden of deaths and economic losses related to PM_2.5_ may further increase, posing a heavy burden on the medical service system [17,18].

This study leveraged advanced monitoring techniques to procure PM_2.5_ concentration data of high spatial resolution, thereby ensuring detailed and granular insights into the air quality across various districts of Beijing. By harnessing the global exposure mortality model (GEMM), this study meticulously estimated the mortality burden that can be directly attributed to PM_2.5_ pollution over a six-year period from 2016 to 2021. This model is renowned for its robustness and enhanced predictive accuracy, which is crucial for quantifying the public health effects of exposure to particulate matter. Further enriching the scope of the research, this study integrated the statistical value of life (VSL) approach—a method that assigns a monetary value to the prevention of fatalities, thereby providing a quantifiable economic perspective on health outcomes. By applying the VSL method, the research was able to compute the economic repercussions stemming from health detriments linked to PM_2.5_ pollution. This economic valuation is particularly significant as it transcends the abstract and oftentimes imperceptible nature of air pollution’s health effects, translating it into a concrete financial framework that can be readily comprehended and acted upon by policymakers and the public alike. The culmination of these methods yielded results that offer a comprehensive and scientifically rigorous foundation for potential policy reform. The findings underscore the critical interplay between air quality and public health and provide an evidentiary basis for the revision of local standards of air quality in Beijing. By considering both the health and economic benefits, this study presents a compelling case for the implementation of more stringent air quality regulations.

## 2. Materials and Methods

### 2.1. Data

This research encompassed an analysis of 16 districts in Beijing to comprehensively assess the repercussions of PM pollution on public health and economic well-being from 2016 to 2021. This study meticulously gathered key data required for this evaluation, emphasizing the significance of data accessibility in this context.

To determine the annual average PM_2.5_ concentration in Beijing from 2016 to 2021, this study relied on the “China Long Time Series with high spatial resolution (1 km × 1 km)” dataset, known as ChinaHigh PMx. The PM_2.5_ data were acquired from research from Donkelaar et al. [19]. This dataset, derived from a combination of various data sources, including land use, air emission, meteorological data, and satellite remote sensing technology, was instrumental in providing a detailed estimation of PM_2.5_ levels [20]. Furthermore, the population data and death data were obtained from the “Beijing 7th National Population Census Main Data Bulletin,” and the insufficient data years were obtained from the Beijing Municipal National Economic and Development Bulletin for 2016–2021 [21]. Additionally, the GDP of each region was obtained from the annual “Statistical Bulletin of National Economic and Social Development,” enabling a comprehensive analysis of economic factors alongside pollution-related data.

We have conducted unified and standardized processing of the data, which included PM2.5 concentration, total resident population, and GDP in the 16 districts of Beijing. All data were unified from 2016 to 2021 to ensure the consistency of the study data. The research results were standardized; that is, the monetary units of the output results of the Beijing VSL model from 2016 to 2021 were unified into US dollars. PM_2.5_ data were intercepted after spatial segmentation by ArcGIS Pro software. The smallest unit was the district-level administrative region, and there were no outliers or missing values.

### 2.2. Mortality Effect Assessment Method

The global exposure mortality model (GEMM) represents a cutting-edge epidemiological framework designed to quantify the health effects of exposure to various risk factors, with a strong focus on air pollution, specifically particulate matter (PM). The GEMM is built upon a rich bedrock of data compiled from a multitude of cohort studies conducted globally. These studies provide extensive empirical evidence on the relationships between exposure to different levels of air pollutants and the resulting mortality risks.

One of the key features of the GEMM is its non-linear exposure–response function, which allows for the estimation of mortality risk even at very low levels of pollution, a domain where many traditional models falter. This is particularly crucial as it enables health impact assessments across a broad spectrum of air pollution concentrations, encompassing both high- and low-exposure scenarios.

The model is adept at evaluating the mortality burden attributable to a variety of air pollutants, for instance, PM_2.5_, ozone, nitrogen dioxide, etc. It takes into account factors such as age, location, and underlying health conditions, which are critical for accurate mortality estimations. The GEMM’s versatility allows for its application in diverse geographical settings and can adapt to varying air quality standards, providing insights that are both locally relevant and globally comprehensive.

The GEMM is an approach to estimating mortality that differs from simpler linear models by providing a more nuanced understanding of the health risks of air pollution. This includes the ability to capture the drastic increase in risk at high pollution levels and the stabilizing of the risk curve at lower levels, which are consistent with the saturation effects observed in epidemiological studies. Quantitative estimates of attributable mortality are based on disease-specific hazard ratio models that incorporate risk information from multiple PM_2.5_ sources (outdoor and indoor air pollution from the use of solid fuels and secondhand and active smoking), requiring assumptions about equivalent exposure and toxicity. It constructed a PM_2.5_-mortality hazard ratio function based only on cohort studies of outdoor air pollution that cover the global exposure range. The GEMM modeled the shape of the association between PM_2.5_ and non-accidental mortality using data from 41 cohorts from 16 countries in the global exposure mortality model (GEMM).

This study assessed the mortality effect of PM_2.5_ based on the exposure–response model and combined it with the PM_2.5_-attributable mortality burden assessment method proposed by Xiao et al. in the Global Burden of Disease study in 2015 [22]. The calculation formulas are as follows:

(1) $$M_{\mathrm{ij}}=\gamma_{0i}\times {\mathrm{AF}}_{\mathrm{ij}}\times {\mathrm{POP}}_{j}$$

(2) $${\mathrm{AF}}_{\mathrm{ij}}=\frac{\mathrm{RR}-1}{\mathrm{RR}}$$

where i represents the endpoints of different health effects, and j represents the grid. $M_{\mathrm{ij}}$ represents the excess deaths attributable to PM_2.5_. $\gamma_{0i}$ refers to the baseline mortality rates for different diseases. ${\mathrm{POP}}_{j}$ is the exposed population of each grid. ${\mathrm{AF}}_{\mathrm{ij}}$ represents an attribution score. RR is the relative risk degree. This study was estimated by the GEMM.

The GEMM is an exposure–response model proposed by Burnett’s team in 2018 to evaluate the health effects of PM_2.5_ [9]. This model is the first exposure–response model to consider the Chinese population and cover the global PM concentration exposure range (2.4–84.0 μg/m). The calculation formulas are as follows:

(3) $$\mathrm{GEMM}\left(\Delta z_{i}\right)=\exp\left\{\left.\beta Τ(\Delta z_{i}|\alpha ,\mu ,\nu )\right\}\right.$$

(4) $$T(\Delta z_{i}|\alpha ,\mu ,\nu )=\log\left(1+\Delta z_{i}/\alpha \right)/\left\{\left.1+\exp\left[-\left(\Delta z_{i}-\mu \right)/\nu \right]\right\}\right.$$

(5) $$\Delta z_{i}=\max\left(0,z_{j}-z_{\mathrm{cf}}\right)$$

In the formulas, z represents the average annual PM_2.5_ concentration for each grid. $z_{\mathrm{cf}}$ refers to the counterfactual minimum PM_2.5_ concentration, which assumes that at this concentration, PM_2.5_ has no effect on health and takes a value of 2.4 μg/m. β represents the exposure–response model coefficient. $T(\Delta z_{i}|\alpha ,\mu ,\nu )$ is a complex transformation function for concentration, where the $\left(\alpha ,\mu ,\nu \right)$ parameter defines the quantitative relation between PM_2.5_ and mortality. According to the research results of Burnet et al. [9], there are two models in the GEMM model that quantify the premature deaths due to PM_2.5_, namely GEMM NCD+LRI and GEMM 5-COD. The former can comprehensively assess the health effects of all non-communicable diseases and respiratory infections induced by PM_2.5_ pollution, as the latter can separately assess five related chronic diseases that are closely linked to PM_2.5_ pollution. These include ischemic heart disease (IHD), stroke (STR), chronic pulmonary obstruction (COPD), lung cancer (LC), and lower respiratory tract infections (LRIs). The GEMM model was utilized to calculate the impacts of variations in PM_2.5_ concentrations on the endpoint of population health effects. We selected the GEMM 5-COD model to study the health effects of 5 causes of death, including ischemic heart disease (IHD), stroke (STR), chronic pulmonary obstruction (COPD), lung cancer (LC), and lower respiratory tract infections (LRIs), caused by PM_2.5_ pollution. Different health responses to atmospheric PM_2.5_ in 12 variant age groups were quantified as well.

### 2.3. Health Economic Benefit Evaluation Method

The VSL (value of statistical life) is a methodological method used in the field of health economics and environmental economics to quantify the monetary value associated with reducing the risk of death. This concept is particularly utilized in cost-benefit analyses where it is essential to weigh the costs of implementing safety measures against the expected benefits of reducing risks to humans’ health. The VSL model is not about the value of an individual’s life per se but rather the value that a group or society places on marginal changes in the risk of death. For example, if individuals in a population are willing to pay a certain amount, collectively, for a reduction in mortality risk that results in one fewer death among them, this amount can be considered the VSL. To calculate VSL, data on individuals’ risk preferences are gathered, often through labor market studies, where wage differentials are analyzed based on job risk levels. These data are then used to infer the amount of money people are willing to pay for small decreases in their probability of death, often termed the “willingness to pay” (WTP) to reduce risk. Essentially, if a population is willing to pay a certain aggregate amount for a measure that reduces the risk of death, that amount can be divided by the changes in the death toll to estimate the VSL.

Using the widely adopted statistical life value method and focusing on cities, this study further assesses the health economic losses attributable to PM_2.5_ pollution [23,24]. It takes the research findings from Beijing in 2012 as the baseline statistical life value. Through the unit value transfer method, the statistical life value for Beijing from 2016 to 2021 is estimated [25].

(6) $${\mathrm{VSL}}_{k,t}={\mathrm{VSL}}_{\mathrm{base}}\times {\left(\frac{G_{c,t}}{G_{\mathrm{base}}}\right)}^{\beta }\times {\left(1+\%\Delta P_{c}+\%\Delta G_{c}\right)}^{\beta }$$

in which ${\mathrm{VSL}}_{k,t}$ stands for the amended VSL value of city c in year t. The VSL for Beijing in the baseline year of 2012 is set at USD 132,000, approximately RMB 936,000 [14]. $G_{c,t}$ stands for the urban GDP per capita in year t, while $G_{\mathrm{base}}$ refers to the baseline per capita GDP, which is the 2012 per capita GDP of Beijing. The income elasticity coefficient is set at 0.8, following the recommendation of the Organisation for Economic Co-operation and Development (OECD) [26]. $\%\Delta P_{c}$ is the factor that accounts for the percentage change in the consumer price index (CPI), and $\%\Delta G_{c}$ is the per capita GDP from the baseline year to year t for city c. By multiplying the calculated result with the deaths linked to PM_2.5_, the total health economic burden for a city in a given year can be determined as follows:

(7) $$E={\mathrm{VSL}}_{c,t}\times M_{\mathrm{ij}}$$

## 3. Results

### 3.1. Analysis of PM_2.5_ Concentrations in Various Districts of Beijing

**Figure 1:**
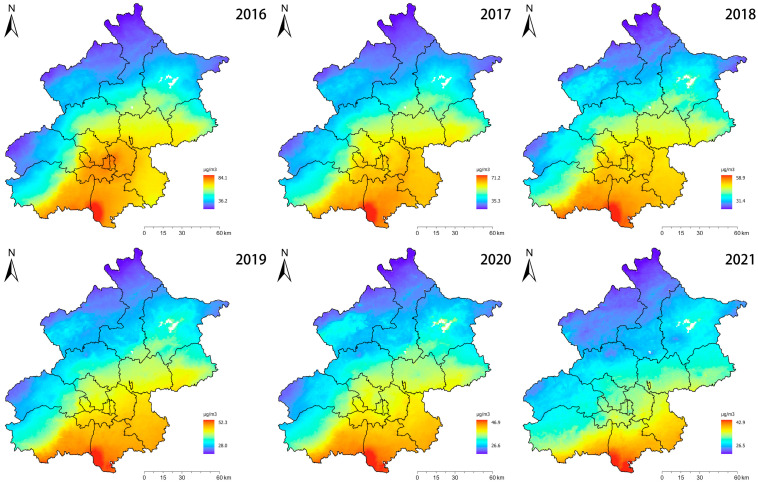
Spatial layout of PM_2.5_ concentrations in 16 districts in Beijing from 2016 to 2021.

According to Figure 1 and Table 1, from 2016 to 2021, there is a clear downward trend in all areas of Beijing. Some regions start with higher values and then decline at different rates. For example, in 2016, Dongcheng District had the highest PM_2.5_ concentration at 75.26 μg/m^3^. By 2021, the maximum concentration becomes 38.90 μg/m^3^ in the Daxing zone. The Dongcheng District decreased from 75.26 μg/m^3^ in 2016 to 35.07 μg/m^3^ in 2021, a decrease of 53.4%. The Huairou region decreased from 47.06 μg/m^3^ in 2016 to 29.74 μg/m^3^ in 2021, a 36.8% decrease, although the Huairou region consistently maintained low PM_2.5_ aggregation levels. The PM_2.5_ concentration in central areas (such as Dongcheng and Xicheng) decreased significantly from a high level, elaborating that the measures of urban air quality improvement have had a significant effect. Although the initial concentration in suburban areas (e.g., Huairou and Miyun) was low, it also showed a steady downward trend.

From the perspective of space, over the years, the districts with high PM_2.5_ aggregation levels mainly located in the central urban area of Beijing and some areas in the south, including Haidian District, Dongcheng District, Xicheng District, Fengtai District, along with parts of Fangshan District and Daxing District. The central region has implemented a series of policies and measures to reduce pollutant emissions and improve air quality, including promoting clean energy, limiting industrial emissions, and raising vehicle emission standards, which have played an important role in reducing PM_2.5_ concentration. According to Table 1, Gu’an City, Hebei Province, also has a higher value of PM_2.5_ concentration, and the aggregation level is similar to that of the central city. The government can promote public transport, encourage the use of low-emission vehicles, reduce vehicle exhaust emissions, and reduce the negative impact of traffic on air quality. Increase the use of clean energy (such as wind and solar energy), reduce the dependence on traditional high-polluting energy, and fundamentally reduce pollutant emissions.

### 3.2. Deaths and Health Related to PM_2.5_ Pollution

Using PM_2.5_ exposure levels and population data from Beijing, combined with the GEMM model, the numbers of premature deaths attributable to PM_2.5_ from 2016 to 2021 were estimated. In 2016, Chaoyang District had the highest number of premature deaths at 13,590, which fell to 7833 by 2021, a reduction of 42.4%. Apart from Chaoyang, Haidian and Fengtai Districts also saw significant decreases in premature deaths, dropping from 12,346 to 6884 and from 8156 to 4547, respectively. A stable downward trend in premature deaths was observed across all districts, aligning with the decreasing trend in PM_2.5_ concentrations. Urban areas such as Dongcheng, Xicheng, and Chaoyang experienced larger declines in premature deaths, possibly due to their denser populations and higher initial pollution levels. Suburban areas like Huairou, Miyun, and Yanqing, despite having lower initial death counts, also showed a downward trend, indicating overall improvements in air quality. The data from all districts highlight the diminishing negative effect of PM_2.5_ pollution on health (Table 2). Reducing PM_2.5_ exposure and translating into improved health outcomes, the first step is to reduce the concentration of PM_2.5_ in the air by reducing the sources of PM_2.5_ emissions, including industry, traffic, and other pollution sources. We will reduce traffic congestion and exhaust emissions by rationally planning urban layouts, increasing green coverage, and improving transportation systems.

**Figure 2:**
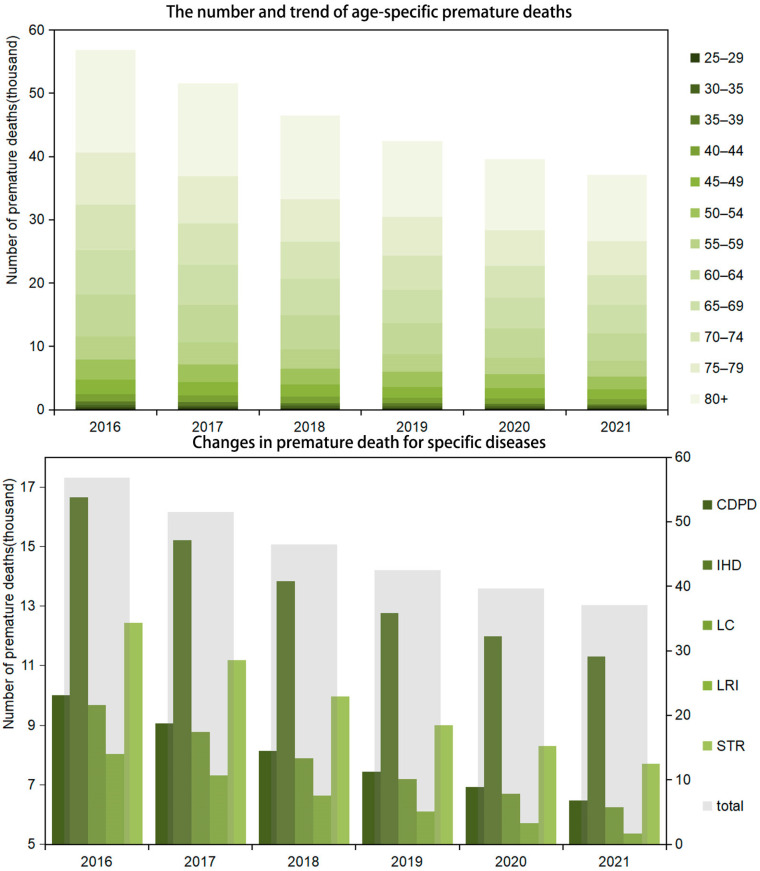
The trend of premature deaths attributed to PM_2.5_ in Beijing from 2016 to 2021 (IHD, ischemic heart disease; LC, lung cancer; LRI, lower respiratory infection; STR, stroke; COPD, chronic obstructive pulmonary disease).

There is a consistent decrease in the figures of premature deaths caused by PM_2.5_ pollution across all age groups over the six-year period. The range of decrease across the age groups is relatively uniform, with the percentage change varying from approximately −32.24% for the 25–29 age group to −35.21% for the 80+ age group. The younger age groups (25–29 to 40–44) have shown a slightly lower percentage decrease in premature deaths compared to the older age groups. The middle age groups (45–49 to 60–64) also follow the decreasing trend but are in the middle range of the percentage decrease. The older age groups (65–69 to 80+) have the highest percentage decrease in premature deaths caused by PM_2.5_ pollution (Figure 2). PM_2.5_ pollution is associated with various diseases. There has been a notable decrease in the number of deaths from these conditions. Specifically, IHD-related deaths decreased from 16,648 to 11,297 over the analyzed period. This significant drop could be attributed to both enhanced medical treatments and interventions and a reduction in exposure to PM_2.5_ pollution, which is a known one of the essential heart disease risk factors. Deaths from lung cancer (LC) attributed to PM_2.5_ pollution fell from 9691 in 2016 to 6253 in 2021. This decline reflects the potential impact of public health initiatives aimed at reducing pollution and smoking alongside advancements in cancer treatment and early detection. Lower respiratory infection (LRI) deaths decreased from 8029 to 5366, underscoring the importance of cleaner air in preventing respiratory infections, particularly among vulnerable populations such as the elderly and children. Stroke-related deaths saw a reduction from 12,443 to 7701 (Figure 2).

### 3.3. PM_2.5_ Health and Economic Benefits

According to Table 3, the overall trend across most districts indicates an increase in the VSL linked to PM_2.5_ pollution over the six years, reflecting a growing economic cost of health impacts led by PM_2.5_. Dongcheng District, Chaoyang District, Haidian District, and Shijingshan District show significant upward trends, with Dongcheng District and Chaoyang District experiencing the most substantial increases. Tongzhou District and Shunyi District show relatively stable or decreasing trends towards the end of the period, suggesting possible improvements in air quality, effective pollution control measures, or changes in the demographic or economic makeup affecting the valuation of health impacts.

**Figure 3:**
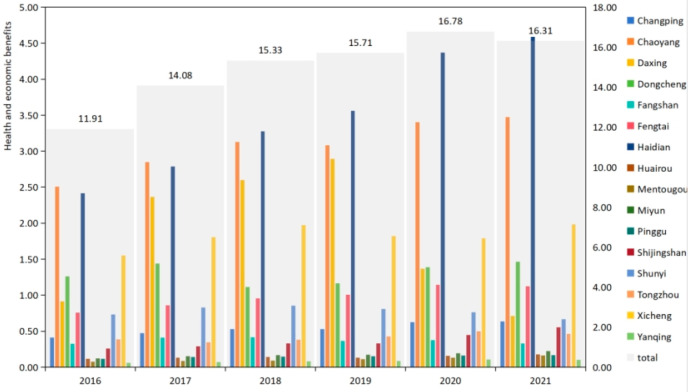
Health and economic losses caused by PM_2.5_ pollution in 16 districts of Beijing from 2016 to 2021 (billion USD).

In Figure 3, the overall trend indicates an increase in the economic losses due to PM_2.5_ pollution, with the total rising from USD 11.91 billion in 2016 to a peak of USD 16.78 billion in 2020 before slightly decreasing to USD 16.31 billion in 2021. Notably, different districts exhibit varying patterns of change, with some districts showing a consistent increase, others experiencing fluctuations, and a few witnessing a decrease in the later years. Districts like Haidian and Chaoyang have shown a consistent increase in losses over the years. These areas are significant economic and educational hubs, implying higher population densities and potentially more vehicular and industrial emissions contributing to pollution levels, thereby increasing health and economic costs. Daxing presents a notable fluctuation, with a sharp increase from 2016 to 2019, followed by a significant decrease. This could be attributed to specific local industrial activities or infrastructural developments, such as the construction and subsequent operation of the Beijing Daxing International Airport, which might have initially increased pollution levels before mitigation measures were implemented. Shunyi district and Daxing district, after initial increases, show a decrease in losses in the later years. The Pandemic since 2020, which caused the closure of Daxing Airport, has resulted in a significant loss of GDP and, consequently, a reduction in the value of statistical life. From the perspective of urban areas, Haidian District and Chaoyang District account for most of the PM_2.5_-related health economic losses in Beijing, and the growth rate and total amount of Haidian District are significantly higher than other districts. Chaoyang District grew at a steady rate between 2016 and 2021. Daxing District had a fast growth rate between 2016 and 2018, but it has decreased significantly since 2019. At the same time, the health and economic losses related to PM_2.5_ in Xicheng District are also high but have always remained in a relatively stable state.

## 4. Discussion

### 4.1. The Attributable Disease Burden of PM_2.5_ in Beijing Showed a Decreasing and Stable Trend

This study focuses on the atmospheric heavy pollution area of Beijing. By using PM_2.5_ data with high-resolution, the international mainstream GEMM method was used to comprehensively evaluate the atmospheric PM_2.5_-attributed deaths and time trends of five causes (COPD, IHD, LC, LRI, and STR), twelve age groups (25–29, 30–35, 35–39, 40–44, 45–49, 50–55, 55–59, 60–64, 65–69, 70–74, 75–79, and 80+), and the total numbers of deaths in Beijing from 2016 to 2021.

In 2012, the NAAQS (the Chinese National Ambient Air Quality Standard), initiated by the Ministry of Environmental Protection of China, listed PM_2.5_ as one of the six pollutants monitored and assessed by AQI [27]. Subsequently, a series of relevant documents have also introduced practical prevention and control actions to improve the air quality in Beijing [28]. The results of this study show that the atmospheric PM_2.5_ concentration in Beijing decreased significantly from 2016 to 2021, but it was still higher than all the national and international standards, including the national secondary standard, the national primary standard, and the WHO guideline standard. Although the attributable death burden of PM_2.5_ in Beijing remains high, due to the improvement in air pollution in Beijing in recent years, the PM_2.5_-attributable deaths of five diseases in 2021 have decreased to a certain extent compared with 2016.

### 4.2. Comparison with Other Studies concerning PM_2.5_-Attributed Deaths and Economic Losses

Compared with previous studies on the disease burden of long-term exposure to PM_2.5_, the advantage of this study is that it uses remote sensing PM_2.5_ data with 1 km high spatial resolution and divides them by district level. At the same time of refinement, the spatial heterogeneity of PM_2.5_ concentration and exposed population was also considered to a great extent. In terms of the total number of deaths, we updated the excess deaths and economic losses attributable to PM_2.5_ in the Beijing area of China based on the innovative GEMM model, based on the calculation of different age groups and the number of deaths.

This study deepens the knowledge of the impacts of PM_2.5_ pollution, consistently indicating that PM_2.5_ pollution represents a severe public health issue, leading to a significant mortality burden [29,30,31,32]. PM_2.5_ poses a serious health threat as their small size allows them to penetrate deep into the human respiratory system. Beyond its direct effects on human health, PM_2.5_ pollution also has profound economic impacts, resulting in substantial economic losses. These losses stem not only from increased healthcare costs but also from indirect factors such as sick leave and decreased productivity. Estimating the economic and health losses is a complex process involving the assessment of the statistical value of life, measurement of pollution levels, the degree of population exposure, and the size of the affected population. The statistical value of life, which represents how much money people are willing to pay to reduce the risk of death, varies across different studies due to cultural, economic, and social values. Similarly, factors like pollution concentration, exposure levels, and population size can vary by region, time, and research methodology, leading to inconsistencies in the estimated economic health losses. All in all, PM_2.5_ pollution not only poses a grave threat to human health but also causes significant economic losses. Addressing this challenge requires global cooperation and comprehensive strategies, including reducing emissions, raising public health awareness, and strengthening policy implementation to alleviate the health and economic burdens of PM_2.5_ pollution. Furthermore, the methods for assessing economic health losses need further research and standardization to more accurately reflect the true impact of PM_2.5_ pollution and provide a scientific basis for policymaking. For example, Maji et al. [33] estimated that the total economic loss related to pollution in China in 2016 was about USD 101.39 billion, accounting for 0.91% of GDP. For example, Maji et al. [33] estimated that China’s total pollution-related economic loss in 2016 was about USD 101.39 billion, accounting for GDP. Among them, the PM_2.5_-related death toll in Beijing was 18.58 thousand, and the economic loss was USD 4.75 billion. This is slightly lower than the number of related deaths (73.92 thousand) and economic losses (USD 11.91 billion) in 2016 in this study. This is mainly because the statistical value of life in Beijing at the time assessed by the study was much lower than that estimated in this study. Han et al.’s findings suggest that economic losses attributable to long-term PM_2.5_ exposure in the eastern region, where Beijing is located, were as high as USD 275.6 billion per year during 2015–2019 [34]. Meanwhile, Xie et al. showed that without effective control measures, it is estimated that by 2030, PM_2.5_ pollution will lead to RMB 210 billion of health expenditure and RMB 10 trillion of health economic losses in China [31]. Previous studies have confirmed that the improvement in air quality in China in recent years has brought considerable health benefits [34,35,36]. Therefore, it is necessary to further take effective air pollution control measures, which will also bring considerable economic benefits. For example, the study by Xu et al. simulated the air quality benefits of the Air Pollution Prevention and Control Action Plan in Beijing in the middle of its implementation and found that the implementation of the policy could reduce external costs by USD 120 million in 2014–2016, which is significant [35].

### 4.3. Policy Implications

In recent years, new changes have taken place in China’s air pollution situation. In order to meet the management needs of ambient air quality in the new era and coordinate environmental protection and economic development, it is necessary to revise the current ambient air quality standards in China [36]. It is worth noting that the formulation and implementation of local air quality standards is of great significance to improve the scientific and precise level of air pollution prevention and control. For example, the US state of California has formulated a series of air quality standards and pollution prevention and control policies based on local conditions, in particular strict control of important pollution sources such as motor vehicle emissions, and has made important progress in improving air quality, energy conservation, and emission reduction and ensuring people’s health [37,38,39]. Beijing is one of the regions with the largest population density and the most active political and economic activities in China, and it is also a key area for air pollution prevention and control in China. Since the release of the “The Atmospheric Ten”, the air quality in Beijing has been greatly improved. Taking Beijing as a pilot city, the formulation of China’s local air standards for PM_2.5_ will help promote the transformation and upgrading of local economic growth models, industrial structures, and technologies and promote the use of clean energy and green economic development to form a low-carbon policy guided by carbon neutrality goals [39,40,41].

Beijing has implemented stringent air pollution control measures over the years, including restrictions on coal-fired power plants, vehicle emission standards, and promotion of green energy, which could explain the overall trend of increasing losses peaking in 2020, as the cumulative effect of pollution on health becomes more pronounced before policies start to significantly mitigate these effects [42]. The economic development in certain districts can lead to increased construction, traffic, and industrial emissions, contributing to higher PM_2.5_ levels and associated health costs [43]. Conversely, economic shifts towards less polluting industries or the relocation of heavy industries out of urban centers can reduce pollution levels [44]. Growing public awareness about the health impacts of PM_2.5_ pollution, coupled with improvements in healthcare services, might influence the economic valuation of health losses as more people seek treatment and adopt preventive measures [29].

In order to further reduce the emission of air pollutants such as PM_2.5_ and continuously improve air quality, it is recommended to adopt a series of regional pollution source control measures. In terms of industrial emissions, it is recommended to establish more stringent emission standards and encourage enterprises to adopt clean energy and efficient purification technologies. At the same time, the promotion of new energy vehicles and the improvement of public transport systems should be accelerated to effectively reduce traffic emissions. For the dust in the construction process, it is recommended to strictly manage and promote green construction standards. In addition, measures such as strengthening public environmental awareness education and expanding urban green areas will encourage the public to adopt a green lifestyle.

It is also crucial to further improve air quality monitoring and early warning systems, increase investment in environmental governance, implement differentiated policies, and improve public health services. It is recommended to encrypt air quality monitoring sites to improve the accuracy and timeliness of monitoring data and to release health warning information in a timely manner in combination with meteorological forecasting to reduce public exposure risks. It is recommended to increase investment in environmental technology research and development, support scientific and technological innovation in clean energy and pollution control, and invest in improving environmental infrastructure. According to the pollution characteristics and economic development level of different regions, it is suggested to formulate differentiated environmental protection policies and strengthen environmental governance cooperation among regions. Through strengthening health education and improving the level of medical services, public health services should be improved so as to provide more comprehensive health protection for the public. These recommendations are designed to continuously improve air quality and public health while also helping to mitigate economic losses and jointly build a greener and healthier future.

While this study underscores the importance of stringent local air quality standards and pollution control measures, implementing these policies faces several challenges. Enforcement mechanisms must be robust and adaptable to ensure compliance across diverse sectors. Engaging stakeholders, including industry, local communities, and policymakers, is essential for fostering a collaborative approach to air pollution mitigation. Moreover, the allocation of resources for monitoring, enforcement, and public awareness campaigns is critical for the success of these measures. Drawing on lessons from cities that have successfully improved air quality, such as Tokyo and London, can provide valuable insights into overcoming these challenges.

### 4.4. Limitations, Uncertainties, Feasibility, Cost-Effectiveness, and Unintended Consequences in Estimating PM_2.5_-Attributed Deaths

This study presents a comprehensive analysis of PM_2.5_ pollution in Beijing and its attributed mortality burden. However, it is crucial to acknowledge the inherent limitations and uncertainties associated with our data and methodology. The estimation of PM_2.5_-attributed deaths relies on the global exposure mortality model (GEMM), which, while robust, is subject to uncertainties related to exposure assessment, population susceptibility, and the transferability of risk estimates across different populations. Additionally, spatial and temporal variations in PM_2.5_ concentrations within Beijing may lead to underestimation or overestimation of exposure in certain districts. Future research should aim to refine exposure assessment methods and explore the heterogeneity of health impacts within urban populations.

The feasibility and cost-effectiveness of proposed pollution control measures are paramount for their successful implementation. Policies must balance environmental benefits with economic impacts, particularly on industries and employment. For instance, transitioning to cleaner energy sources requires significant investment and infrastructure development. Additionally, unintended consequences, such as the displacement of pollution to neighboring regions or increased financial burdens on low-income households, must be carefully considered. Mitigation strategies, including economic incentives for clean technologies and targeted support for affected communities, can help address these issues.

## 5. Conclusions

This study meticulously gathered data on the annual average concentrations of permanent residents, GDP, and PM_2.5_ across 16 districts of Beijing from 2016 to 2021. Utilizing the GEMM method, we calculated the deaths caused by PM_2.5_ pollution, which showed a fluctuating yet concerning trend with numbers like 9157 in 2016, peaking at 12,723 in 2020 and reducing slightly to 12,352 in 2021. These data provide a stark explanation of the impact of PM_2.5_ pollution on people’s health. Further, this study evaluated the corresponding health economic losses using the health economic benefit evaluation method, revealing staggering economic impacts ranging from USD 9232.27 million in 2016 to USD 12,735.42 million in 2021.

The PM_2.5_-pollution-related economic losses fluctuated across 16 districts from 2016 to 2021, illuminating the intricate relationship between urban development, environmental policies, and public health measures. While there is growing public awareness of the impact of air pollution on population health and economic development, changes and differences at the district level point to the need for a more nuanced approach to pollution prevention and health risk management. Given the serious mortality burden and economic losses attributed to PM_2.5_ exposure in Beijing, it becomes imperative to reevaluate and tighten the annual average standards for PM_2.5_ air quality. The current situation demands a more scientific, precise, and localized approach to pollution prevention and control tailored to the evolving dynamics of atmospheric pollution. Establishing more rigorous and comprehensive local environmental air quality management standards is crucial for Beijing. Such measures should not only aim at meeting but surpassing the national standards, taking into account the specific challenges and needs of the city’s diverse districts.

There is a close relationship and influence between air pollutants and building disciplines. Air pollutants will have an impact on building materials, the indoor environment, building energy efficiency, and other aspects, and have an impact on the indoor environment. In addition, air pollutants also have a certain impact on building energy conservation. The architectural discipline fully considers the harm and impact of air pollutants in the design and construction process, improves the comfort and health level of the indoor environment, and realizes the green, low-carbon, and sustainable development of buildings. Beijing’s experience underscores the critical importance of sustained, comprehensive strategies that balance economic growth with environmental sustainability and public health. The path forward requires not just stringent standards but a collective effort to transform awareness into action, ensuring a healthier future for all residents.

## Figures and Tables

**Table 1:** PM_2.5_ concentrations in 16 districts in Beijing from 2016 to 2021 (μg/m^3^).

	2016	2017	2018	2019	2020	2021
Changping	58.17	50.26	43.76	37.32	34.33	31.83
Chaoyang	72.75	60.65	50.84	44.46	39.48	35.54
Daxing	73.97	64.34	53.75	47.79	42.83	38.90
Dongcheng	75.26	60.74	50.93	44.42	39.08	35.07
Fangshan	62.18	54.91	48.03	42.18	38.22	34.98
Fengtai	73.56	61.07	51.65	45.13	40.33	35.19
Haidian	68.64	57.52	48.98	41.92	38.22	33.80
Huairou	47.06	43.30	38.03	33.47	31.04	29.74
Mentougou	49.22	46.25	41.20	35.78	32.99	31.66
Miyun	50.74	46.19	40.64	36.35	32.98	31.54
Pinggu	61.18	53.64	45.38	40.96	36.44	33.72
Shijingshan	71.72	59.63	50.87	43.25	39.47	34.48
Shunyi	66.22	57.05	48.15	42.20	37.74	34.05
Tongzhou	69.04	61.57	51.20	46.08	41.19	37.73
Xicheng	75.75	60.45	50.97	44.11	38.94	35.09
Yanqing	45.17	42.89	38.22	33.38	31.41	29.57

**Table 2:** Number of deaths related to PM_2.5_ pollution in 16 districts of Beijing, 2016–2021.

	2016	2017	2018	2019	2020	2021
Changping	6281 (4805–7527)	5897 (4493–7100)	5561 (4225–6724)	5154 (3906–6258)	5044 (3818–6136)	4801 (3632–5850)
Chaoyang	13,590 (10,468–16,161)	11,980 (9175–14,337)	10,523 (8022–12,667)	9365 (7118–11,319)	8388 (6362–10,170)	7833 (5932–9522)
Daxing	5971 (4602–7097)	5750 (4411–6867)	5333 (4070–6408)	5194 (3954–6264)	5098 (3872–6168)	4799 (3639–5821)
Dongcheng	3065 (2364–3641)	2618 (2005–3133)	2259 (1722–2719)	1971 (1498–2382)	1711 (1298–2076)	1594 (1207–1938)
Fangshan	3508 (2688–4194)	3423 (2614–4110)	3264 (2485–3936)	3165 (2403–3830)	3125 (2369–3793)	2951 (2235–3589)
Fengtai	8156 (6284–9695)	7163 (5487–8570)	6298 (4803–7578)	5581 (4243–6742)	4974 (3774–6028)	4547 (3443–5529)
Haidian	12,346 (9492–14,711)	10,817 (8272–12,968)	9519 (7249–11,471)	8312 (6310–10,062)	7458 (5654–9051)	6884 (5210–8377)
Huairou	1087 (827–1312)	1059 (804–1281)	999 (757–1212)	935 (707–1138)	918 (694–1119)	892 (674–1088)
Mentougou	925 (704–1115)	924 (703–1115)	895 (679–1084)	852 (645–1036)	851 (644–1036)	834 (631–1017)
Miyun	1404 (1070–1691)	1342 (1020–1620)	1258 (954–1524)	1189 (901–1444)	1143 (865–1392)	1108 (838–1350)
Pinggu	1357 (1039–1623)	1277 (975–1535)	1176 (894–1421)	1108 (840–1342)	1055 (799–1282)	1003 (759–1221)
Shijingshan	2263 (1742–2693)	1982 (1517–2373)	1750 (1334–2106)	1530 (1162–1850)	1380 (1046–1673)	1260 (954–1533)
Shunyi	3626 (2784–4326)	3498 (2674–4195)	3300 (2512–3979)	3189 (2421–3859)	3127 (2370–3795)	2930 (2218–3565)
Tongzhou	4962 (3816–5912)	4922 (3771–5887)	4672 (3562–5623)	4637 (3527–5599)	4591 (3484–5560)	4349 (3296–5279)
Xicheng	4530 (3494–5380)	3889 (2978–4655)	3416 (2604–4111)	3006 (2284–3633)	2664 (2020–3231)	2487 (1883–3024)
Yanqing	838 (637–1013)	833 (633–1008)	789 (598–958)	732 (554–891)	726 (549–884)	697 (527–851)

**Table 3:** The value of statistical life in 16 districts of Beijing from 2016 to 2021 (×10^4^ USD).

	2016	2017	2018	2019	2020	2021
Changping	6.52	8.01	9.51	10.29	12.35	13.19
Chaoyang	18.44	23.79	29.74	32.93	40.56	44.36
Daxing	15.34	41.10	48.71	55.75	26.81	14.80
Dongcheng	41.07	54.98	49.33	59.13	81.11	91.76
Fangshan	9.27	12.02	12.81	11.52	12.04	11.19
Fengtai	9.26	11.99	15.17	18.00	23.04	24.69
Haidian	19.56	25.76	34.40	42.84	58.56	66.66
Huairou	10.47	12.30	14.23	14.20	17.29	20.04
Mentougou	8.24	9.02	10.11	12.71	15.40	19.23
Miyun	8.64	11.39	13.40	14.37	16.71	20.29
Pinggu	8.64	11.02	12.52	13.51	15.08	16.67
Shijingshan	11.50	14.64	18.88	21.57	32.42	43.77
Shunyi	20.17	23.70	25.87	25.35	24.34	22.64
Tongzhou	7.79	7.05	8.12	9.15	10.81	10.63
Xicheng	34.17	46.41	57.76	60.62	67.12	79.81
Yanqing	6.89	8.62	10.38	11.66	14.23	14.52

## Data Availability

Data are contained within the article.
